# 3D geological implicit modeling method of regular voxel splitting based on layered interpolation data

**DOI:** 10.1038/s41598-022-17231-x

**Published:** 2022-08-16

**Authors:** Jian Li, Peirong Liu, Xinyu Wang, Hao Cui, Yurong Ma

**Affiliations:** 1 School of the Geo-Science & Technology, Zhengzhou University, Zhengzhou, 450001 Henan China; 2 School of Water Conservancy Science & Engineering, Zhengzhou University, Zhengzhou, 450001 Henan China; 3 University Library, Zhengzhou University, Zhengzhou, 450001 Henan China

**Keywords:** Civil engineering, Geology

## Abstract

In view of the problems in traditional geological modeling methods, such as the insufficient utilization of geological survey data, the inaccurate expression of a stratigraphic model, and the large amount of model data, a 3D geological model cannot be smoothly loaded and rendered on the web end. In this paper, a 3D geological implicit modeling method of regular voxel splitting based on hierarchical interpolation data is proposed. This method first uses the boreholes and geological section data from a geological survey for data conversion and fusion, compares the applicability of different interpolation algorithms through cross-validation research, and uses the best fitting algorithm to interpolate and encrypt discrete points in the formation. Then, it constructs the regular voxels, designs five different regular voxel split types, and divides the voxels. In addition, the data structure design of the voxel split model is implemented, and the irregular voxel metadata structure is analyzed and displayed through Three.js. Using this method, based on the survey data of an area in Zhengzhou, the global workflow from data processing to model construction and visualization is demonstrated. The experimental results show that the model can integrate multisource hierarchical interpolation data; express different stratum structures accurately and smoothly, and can realize the rendering, spatial query and analysis of the internal information of a geological body in a browser.

## Introduction

In the process of the development of urban modernization, the investigation and analysis of geological resources and the development and utilization of underground space play important roles^1–7^. As an effective tool to improve the efficiency of underground space surveying, management and analysis, an accurate and meticulous 3D geological model can directly display the geological structure, lithology, spatial shape and other attributes of strata and provide accurate information for professionals to analyze geological structures and fault distribution, which can provide a reliable basis for underground space analysis and decision-making^8–12^.

The primary difficulty of 3D geological modeling technology is the use of limited and discrete geoscience data to accurately express complex geological bodies. Traditional modeling methods often use a single data source, and the constructed models cannot support an accurate and complete 3D solid representation. To solve this problem, Hademenos^13^ and Graciano^14^ add a geological profile and a seismic profile on the basis of borehole data and reduce the uncertainty of the stratum information between boreholes after integrating geologists' understanding of geological phenomena. Jørgensen^15^ and Høyer^16^ combine borehole data with airborne electromagnetic (AEM) data, obtain dense spatial information via AEM, and provide lithologic information through the measured resistivity. To enrich the data and establish a fine and reliable data model, Høyer^17^ fuses geological maps, boreholes, near-surface electromagnetic induction (EMI), geological profiles and TEM5 data together and analyzes the functional characteristics of different data in subsequent analysis and application. The combination of geophysical, geomorphological and geological information provides sufficient data support for the research and analysis of underground three-dimensional spaces, but most of these methods are based on existing mature software, and there is a lack of research on a unified format and mutual fusion of the original data. In addition, based on the fused data, 3D models are often constructed using a single interpolation method, and its spatial distribution function cannot sufficiently fit the topography of the whole region.

The second difficulty of 3D geological modeling is the selection of the 3D spatial data model. As the basis of the visualization of geological modeling, a 3D spatial data model is of great significance for the accurate expression of geological information and the subsequent analysis and application^18^. According to the differences in the expression of spatial data models, spatial data models can be roughly divided into three categories: surface models, voxel models and hybrid models^19^. Of the three types of models, the construction of a surface model is simple and efficient, but it puts too much emphasis on the surface expression of a 3D space, and the attribute information inside the geology is blank; therefore, it is unable support common engineering applications such as groundwater simulation^20^. Although a hybrid model can combine the advantages of different data models and effectively take into account the internal and surface expression of the geological body, due to the complex data structure of the model, it is difficult to implement and update, and the application process involves considerable manual work. Therefore, hybrid models are seldom used in practical engineering. As an extension of the 2D raster data structure in the 3D space, the voxel model divides the 3D space into nonoverlapping and interconnected geometric elements and uses the voxel information as the basic unit to establish the geological model. Different from the spatial shape simulation of geological structure modeling, in the 3D visualization of the internal geological structure stage, the various heterogeneous properties expressed by voxels have prominent engineering significance^21–24^. In addition, this data structure is more convenient for spatial querying, statistics and analysis; therefore, the realization of a 3D geological description using a volume model is attracting increasing interest from the earth science community.

As early as 1987, geological voxels started to be the basis for the establishment of geological 3D models^25^ and gradually developed into regular voxels and irregular voxels according to whether the shape was regular or not. The more commonly used shapes include regular blocks, octrees, generalized triangular prisms (GTP), tetrahedrons and so on. In regular voxels, with the development of 3D GIS, visualization based on regular blocks is widely used in the simulation of metal orebodies since it simplifies the calculation and analysis processes in orebody development^26^. The application of octrees realizes the multiscale digital representation and modeling of a 3D geology^27^, which can meet the requirements for the rapid visualization of large data sets^15^. In the research process of irregular voxels, the tetrahedral model proposed by Victor et al.^28,29^ and Pliout et al.^30^ can achieve efficient geometric transformation and topological relationship processing. The subsequent unstructured tetrahedron model further solves the defect that structured meshes cannot adapt to more complex shapes, and thus is widely used in the finite element analysis of irregular discrete points and complex structure modeling^31,32^. On the basis of the analogical tri-prism model, Wu et al.^22^ proposed the generalized triangular prism data model, and realized 3D geological modeling under the limitation of inclined boreholes.

Most of these methods based on voxel modeling have achieved good results, but they still cannot consider the smooth description of a boundary and the efficient expression of attributes at the same time. For example, the modeling based on regular blocks will cause jagged boundary defects, and the octree model is essentially a compression of the 3D voxel model, which is not conducive to the study of other geological attributes. In addition, as irregular voxels, the GTP model and tetrahedron model have complex data structures and focus on the flexibility of geological simulation, which result in their inability to uniformly express geological bodies^33^. In addition, in the visualization stage of a 3D geological model, most of the current studies rely on desktop applications or browser plug-ins^16,17,34–39^, which reduces the universality of the modeling methods and techniques to a certain extent.

To solve the above problems, this paper proposes a complete process of 3D geological implicit modeling that abandons the tedious process of traditional display modeling and manual delineation and integrates borehole and geological profile data. On the basis of the hierarchical interpolation and encryption of the fused data, this paper combines borehole and geological section data, and proposes a regular voxel splitting model on the basis of the hierarchical interpolation and encryption of the fusion data. The model is based on regular blocks and can judge the relationship between the voxel and the stratum interface in real time during the voxel filling process, and perform different splitting processes on the voxel. The splitting process not only ensures the effective description of the internal attributes of the geological body by the voxel, but it also realizes the accurate display and expression of the stratigraphic boundary. In addition, the data structure designed for the model elements in this paper can achieve efficient data access and analysis in the visualization stage. Based on this process, the prototype system developed using Three.js can eliminate the constraints of desktop applications and can realize the construction and rendering of geological models on the web end without browser plug-ins.

## Hierarchical interpolation data combining borehole and geological section

The 3D geological implicit modeling method proposed in this paper extracts layered nodes using controlled boreholes and then conducts the discrete transformation of the 2D profile, thus unifying the coordinate format of the two kinds of data. Based on the fused data, this paper cross-validates the commonly used interpolation algorithms. In view of the differences in the distribution of discrete points and the structure of the layered interfaces in different strata in the experimental area, the algorithm with the best fitting effect is used to realize the interpolation and encryption of discrete points in strata. This implicit modeling method can calculate the exact location of all data points and provide built-in volume consistency, which is convenient for subsequent 3D geological model construction.

### Fusion of borehole and section data

Boreholes are the most important data source for 3D geological modeling^13,40,41^. However, the acquisition cost of borehole data is high, and the data are limited and sparsely distributed. Borehole data cannot accurately and continuously reflect the actual stratigraphic structure and geological conditions, and it is difficult to meet the requirements of high-precision 3D geological modeling using these data. As another common data source for the construction of complex geological body models, geological sections are relatively rich and continuous, which can well express the horizontal extension of the control area strata, visually display stratigraphic boundaries, and can be used as a data source for 3D geological modeling. However, as 2D images, geological section data cannot be accurately located in 3D space. Therefore, in order to make full use of the limited geological data, it is necessary to combine borehole and geological section data to improve the modeling accuracy. The main work includes the following: the extraction of layered nodes in boreholes and the discretization and conversion of section data.

The spatial distribution of urban underground geological structures is highly uneven, and the results based on lithologic stratification are often unreliable. It is necessary to stratify the boreholes according to their chronological sequence and establish a comprehensive stratigraphic interface of time and lithology in order to establish a 3D geological model with isochronous significance in space and achieve the purpose of accurate modeling.

Controlled boreholes can be used to determine the hierarchical nodes of the age on each borehole. On this basis, considering the stratigraphic lithology, color, formation and other factors, with reference to “The unified division standard of Quaternary strata in Henan Plain”, each borehole is divided into four periods: Holocene (approximately 12,000 years), Upper Pleistocene (approximately 150,000 years), Middle Pleistocene (approximately 730,000 years) and Lower Pleistocene (approximately 2.48 million years). By studying the geological characteristics of the buried depth of the floor in different periods and then matching other boreholes, the Quaternary stratigraphic division is conducted on the rest of the boreholes one by one.

**Figure 1:**
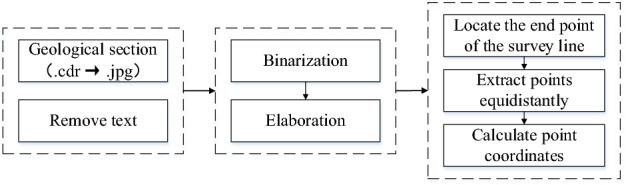
Section information extraction process.

Since the section data are in an image format and cannot be directly fused with borehole data for model construction, it is necessary to perform discrete conversion of the data first to extract the 3D spatial point data required for the construction of the stratum interface. On the basis of image preprocessing, in order to place the extracted discrete points and the borehole layered nodes in the same coordinate system, the section is first registered based on the coordinates of the starting point of the survey line. Then, the extraction spacing needs to be set according to the accuracy requirements, and the points are extracted equidistant from each stratigraphic boundary. The row number of the extraction point is then located, and its coordinates and elevations are calculated. Figure 1 shows the discrete transformation process of the section data. The strata nodes obtained through drilling stratification have the same geological stratification attributes as the strata nodes extracted from the section, and they can be directly used as the basic data for 3D geological modeling.

### Hierarchical interpolation of fused data

The essence of spatial interpolation is to fit a functional relation to approach the sampling point as closely as possible by analyzing the spatial distribution of the original sampling point, and the attribute value of any position in the area can be determined through this functional relation. Under the active exploration and research of many scholars, algorithms such as Kriging interpolation, thin plate spline interpolation and inverse distance weighted interpolation are prominent. These interpolation methods are effective and widely used in practical engineering, but each method has its own applicability. In practical use, it is still necessary to choose flexible and reasonable spatial interpolation methods according to the engineering geological conditions, data characteristics and data volume.

To construct an accurate and detailed 3D geological model, this paper analyzed and compared several classical interpolation algorithms and uses cross-validation to evaluate the actual applicability of each algorithm in various layers. This allowed for making full use of the fused data in the process of constructing the layer point model to realize the fine construction of the 3D geological model.

To verify the stratigraphic applicability of commonly used interpolation algorithms, the exploratory spatial data analysis of each stratigraphic boundary point was conducted to verify the spatial autocorrelation between the data. If there is spatial autocorrelation between the sampling points, the differences in the sampling points with similar distances in space will be relatively small and the similarity will be large; conversely, the sampling points with relatively great distances will have the opposite characteristics. The commonly used autocorrelation measures include the variation function, the covariance, and the Moran index, among others. In this paper, the cloud map of the variation function was used to analyze the spatial autocorrelation.

On the basis of the spatial autocorrelation analysis, this paper used the algorithm with the best degree of fit to interpolate and encrypt the sampling points of each stratum interface; however, because the difference in the actual interpolation results was small, choosing a method based on its visual effect was easily affected by subjective judgment. Therefore, this paper used the leave-one-out method to cross-validate the interpolation results. The first sampling point from the data set containing N samples was selected layer by layer, and the remaining N-1 sample points were used as modeling samples. Next, different interpolation methods were chosen to fit sample No.1 and calculate the error between the fit value and the measured value, then, sample No. 1 was put back into the whole sample set, and another sample that was not included in the modeling was selected. This cycle continued until all the samples were used for a test. The results of cross-validation include the measured values and the fit values.

## Regular voxel split model and data structure design

The core idea of the regular voxel splitting model is based on the regular voxels. In the modeling process, with the filling of the voxel in the geological body, the positional relationship between the voxel and the stratum interface is judged in real time, and the voxel is split differently. That is, when the voxel is cut by the stratum interface, it will be divided into several irregular entities; and if the voxel lies completely within the interface of the two layers, its regular shape will be retained. The experimental results show that this method can compensate for the deficiency of the regular voxel in geological modeling, so that it can express the geological surface smoothly and describe the boundary position accurately.

**Figure 2:**
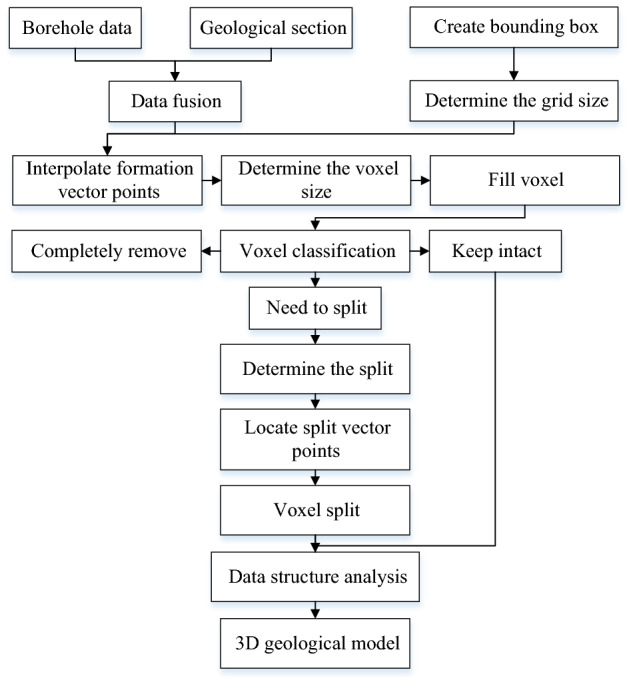
Modeling process.

The modeling method using the regular voxel split model is shown in Fig. 2.

### Voxel classification

**Figure 3:**
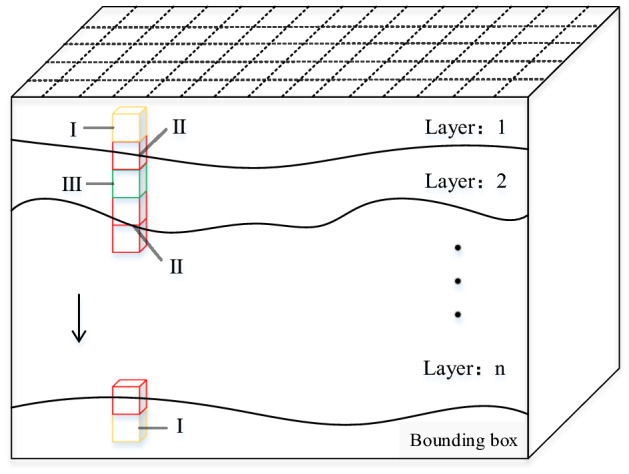
Schematic diagram of voxel filling.

As shown in Fig. 3, when the voxel is filled from the top down inside the bounding box, the voxel can be divided into the following three categories according to the positional relationship between the voxel and the formation interface.

1. Complete removal (type I): Because the bounding box is larger than the actual boundary, the type I voxel generated at the beginning of filling or during the filling to the bottom does not belong to any geological body.
2. Need to split (type II): This kind of voxel is passed through the stratigraphic interface; therefore, it needs to be split, and the entities formed after splitting belong to different geological bodies.
3. Complete retention (type III): The voxels exist completely between two stratigraphic interfaces.

**Figure 4:**
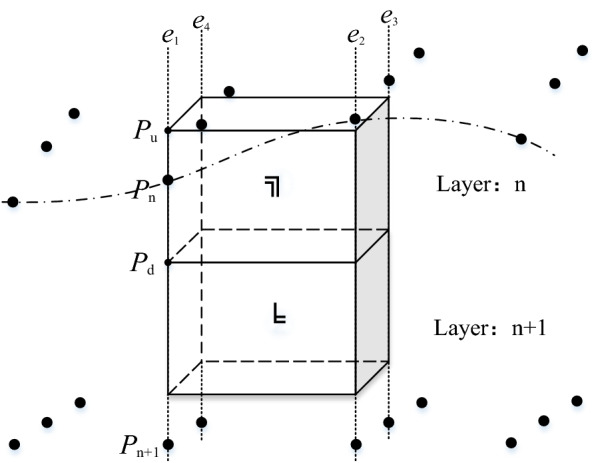
Schematic diagram of voxel positions.

When using the regular voxel splitting model for modeling, accurately classifying all voxels and assigning geological attributes to each voxel is the key to ensuring the reliability of the model. To classify voxels, this paper uses regular grid control to interpolate at each grid point of the stratigraphic interface, and the voxels and stratigraphic points will have a positional relationship, as shown in Fig. 4. When the voxel is traversed by the stratum, there will be vector points on the corresponding edge. We only need to judge whether there is a stratum point on the edge, and the three types of voxels can be judged. In addition, the basic data of modeling all have geographic coordinates, and all the models are constructed are in the same coordinate system. Using the coordinates as a reference can easily judge the spatial positional relationship of voxels.

As shown in Fig. 4, the four edges of the voxel are respectively denoted as *e*_1_-*e*_4_. First, the two endpoint coordinates *P*_1u_ (*x*, *y*, *z*_u_) and *P*_1d_ (*x*, *y*, *z*_d_) of edge *e*_1_ are calculated from the coordinates of the center point of the voxel. Then, the vector points of the stratum interface at the corresponding lattice points are retrieved by abscissa *x* and ordinate *y*, which are marked as *P*_1_ ~ *P*_n_ (n = 6 in this article), and the coordinates are recorded as (*x*, *y*, *z*_u_). Afterwards, by comparing the sizes of *z*_u_, *z*_d_ and *z*_n_, the positional relationship between edge *e*_1_ and the formation interface point is judged, and the positions of the other three edges can be judged using the same method. If there is a vector point on the edge of the voxel, the voxel needs to be split. For example, there are vector points on edges *e*_1_ and *e*_4_ of voxel ① in the figure. If there are no vector points on the four edges of the voxel, the voxel may be removed or retained. Voxel ② in the figure should be retained because it is between two strata.

### Voxel split realization

**Figure 5:**
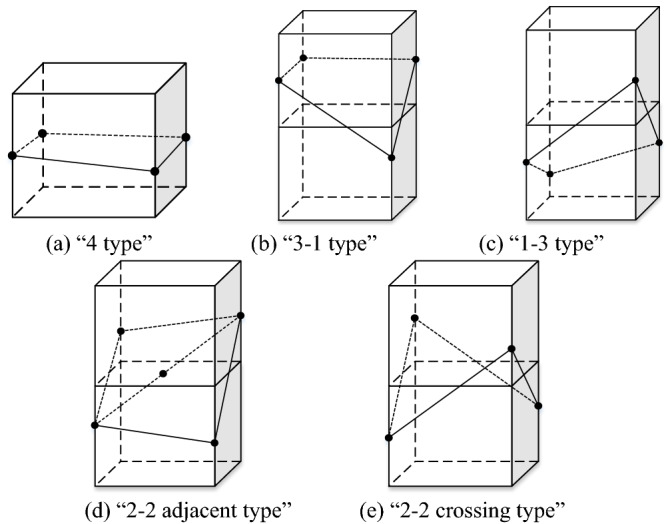
Five types of voxel splits.

Even if they are all split voxels, due to the uncertainty of the stratum interface, the stratum will have different directions and angles when passing through the voxels. In this paper, according to the positional relationship between the upper and lower adjacent voxels and the vector points of the stratigraphic interface, the split voxels are divided into five types: "4 type", "3–1 type", "1–3 type", "2–2 adjacent type" and "2–2 crossing type". Figure 5 takes the "2–2 adjacent type" as an example to illustrate the voxel splitting process.

**Figure 6:**
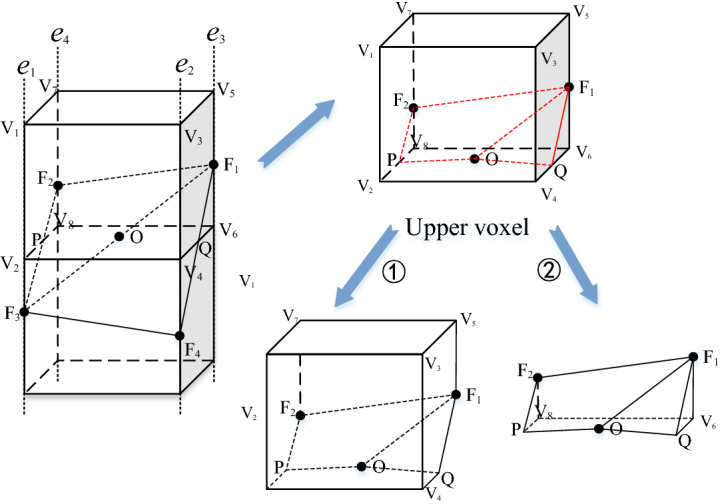
“2–2 adjacent type” upper voxel splitting process.

The flow of the entire splitting process is shown in Fig. 6. In the end, the voxel is split into two irregular entities. Entity ① and entity ② belong to the same voxel, but they are separated by a certain stratum; therefore, that they belong to different geological bodies. In addition, *F*_1_ and *F*_2_ shown in the figure fall on *e*_3_ and *e*_4_, respectively (reflected in Fig. 4). However, because of the uncertainty, it is possible to fall on other edges in other voxels, so locating the region (index) of the edge of *F*_1_ and using it as the benchmark to solve the model can ensure the versatility of the algorithm.

In the figure, *V*_1_ ~ *V*_8_ represent the vertexes of the upper regular voxel, which are obtained by calculating the coordinates of the center of the voxel. *F*_1_ ~ *F*_4_ are the vector points belonging to the same stratum interface, and the coordinates have been calculated during grid interpolation. Points *O*, *P* and *Q* are the unknown points that need to be solved, which are the points of the intersection between *F*_1_*F*_3_, *F*_2_*F*_3_, *F*_1_*F*_4_ and the lower surface of the voxel. In this paper, these points are solved by the vector method in the implementation of the program.

### Data structure design of the voxel splitting model

To make the data organization, management and sharing of the split model more convenient, the model was divided into four basic elements in this paper: points, triangles, regular voxels and irregular voxels. Then, the data structure was designed to realize data storage and analysis.

#### Point

**Figure 7:**
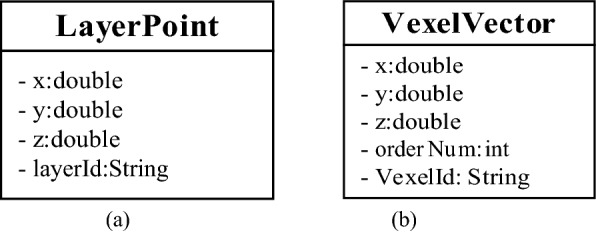
Data structure-point.

In the process of constructing the geological model, two types of point data are produced. One type of point data is the vector points of the stratigraphic interface obtained by the interpolation of boreholes and sections. As shown in Fig. 7a, when designing the data structure, it is necessary to record the sequence number of the stratigraphic interface by using the layerId field in addition to recording the coordinate information (*x*, *y*, *z*). The second type of point data is the vertices of the irregular voxels generated during the split. As shown in Fig. 7b, in order to facilitate model reconstruction and visualization, the orderNum field records the number of each vertex in the voxel and binds the voxel through the vexelId field.

#### Triangle

**Figure 8:**
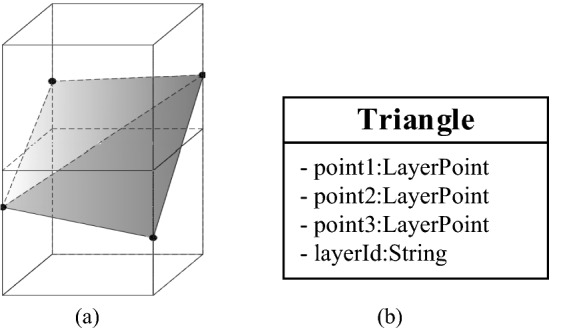
Data structure-triangle.

To achieve different visualization requirements, the organization of data should meet the requirements of the geological entity model and geological surface model at the same time. As shown in Fig. 8a, when the stratum surface passes through two upper and lower adjacent voxels, the four vector points of the same stratum controlled by the regular grid will form two triangular faces on the cutting plane of the voxel. Each triangular plane is composed of three formation vector points called LayerPoints, and all triangular planes of the same formation can be combined into a formation surface model. Figure 8b is a triangular data structure. In addition to recording three vertices, the layerId field is used to identify the interface of the strata to which it belongs.

#### Regular voxel

**Figure 9:**
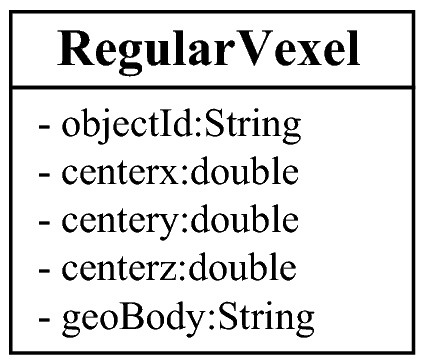
Data structure-regular voxel.

The regular voxel is the most common data model in the modeling process. Its characteristics are that the length and width of the horizontal direction are controlled by the grid, and the height is strictly unified. When visualizing the model, the position of each vertex and surface can be calculated from the central coordinates. As shown in Fig. 9, in order to save storage space, only the central coordinates (*x*, *y*, *z*) of the regular voxel are recorded when storing the regular voxel, and the geological body to which the voxel belongs is recorded using the geoBody field.

#### Irregular voxel

**Figure 10:**
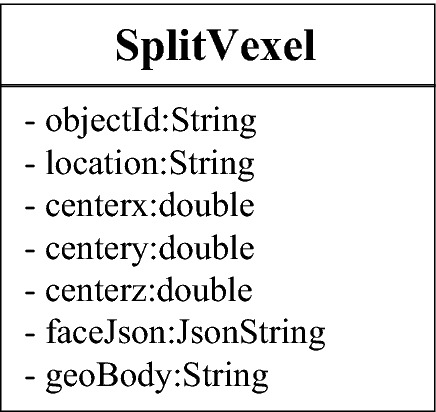
Data structure-irregular voxel.

An irregular voxel is the most complex data model, and the entity shapes formed by the 5 splitting situations are also different. Since the vertex position and each surface of the entity cannot be solved directly, it is necessary to record each vertex and surface at the same time when designing the data structure of irregular voxels. Previously, when designing the data structure of points, the vertices of irregular voxels were stored in VexelVector, and the identification ID of the voxel was bound; therefore, when reading an irregular voxel, all the vertices of the voxel can be retrieved through their IDs. Figure 10 shows the data structure of irregular voxels. Json is a general data exchange format that can be used to store every surface of irregular voxels, and each vertex on the surface is recorded counterclockwise.

### Model realization

**Figure 11:**
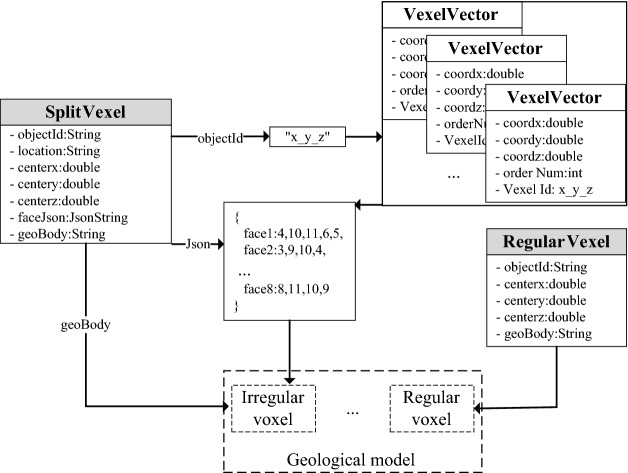
Data analysis process.

The model implementation process is the parsing process of the data structure. How to calculate the shapes, positions and attributes of voxels through the reading of the database and the analysis of the data structure is the core task of the model. As shown in Fig. 11, in the experiment of this article, voxels are divided into two types: regular voxels (RegularVexel) and irregular voxels (SplitVexel). It is easy to analyze a regular voxel, and the positions of the 8 vertices and 6 surfaces can be calculated directly from its central coordinates. When analyzing irregular voxels, we should first retrieve the coordinates and numbers of each vertex according to the identification ID and obtain the vertices contained in each surface by parsing the faceJson field. Then, it is necessary to complete the rendering of each surface of the irregular voxel according to the order and coordinates of the vertices. Finally, the texture (color) is set for each voxel through the geological attribute information in the geoBody field.

## Case application and analysis

Based on the above method, this paper realized the design and development of the geological modeling prototype system based on Three.js for a Windows environment and conducted an experimental study with a certain urban area of Zhengzhou as the research area. A total of 33 geological boreholes were collected, of which 16 were shallow boreholes with a depth of more than 60 m, and the remaining boreholes were all deeper than 100 m. First, 4 controlled boreholes were used for dating, and the other 29 boreholes were divided into Quaternary strata one by one. Through the matching of the geological characteristics, three stratigraphic demarcation points are determined in shallow boreholes, and five stratigraphic demarcation points were determined in deep boreholes.

A total of 8 shallow seismic exploration lines were collected in the experiment, of which 6 segments fell within the study range, and the seismic depth sections of the corresponding sections could be obtained from the original data. First, the original section was preprocessed, the image format was converted, the irrelevant information in the image was removed, and then it was abstracted as a 2D matrix. Then, the threshold was set, and the image was binarized, which was processed into a binary image with gray values of only 0 or 255. Since the width of the stratigraphic boundary is often greater than one pixel, the image was further refined in order to accurately extract layered information based on lines.

**Figure 12:**
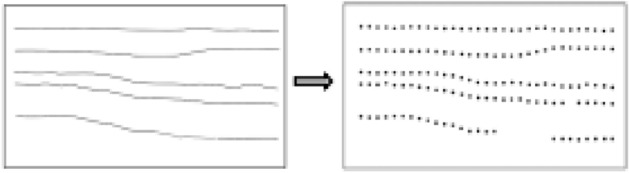
Schematic diagram of section information extraction.

Finally, the section was registered based on the coordinates of the starting point of the survey line. On this basis, it was necessary to count the number of pixels occupied by horizontal and vertical unit scales, calculate the actual distance and depth represented by a single pixel, and then extract points at equal distances from each stratigraphic boundary. Figure 12 shows the results of discrete point extraction.

**Figure 13:**
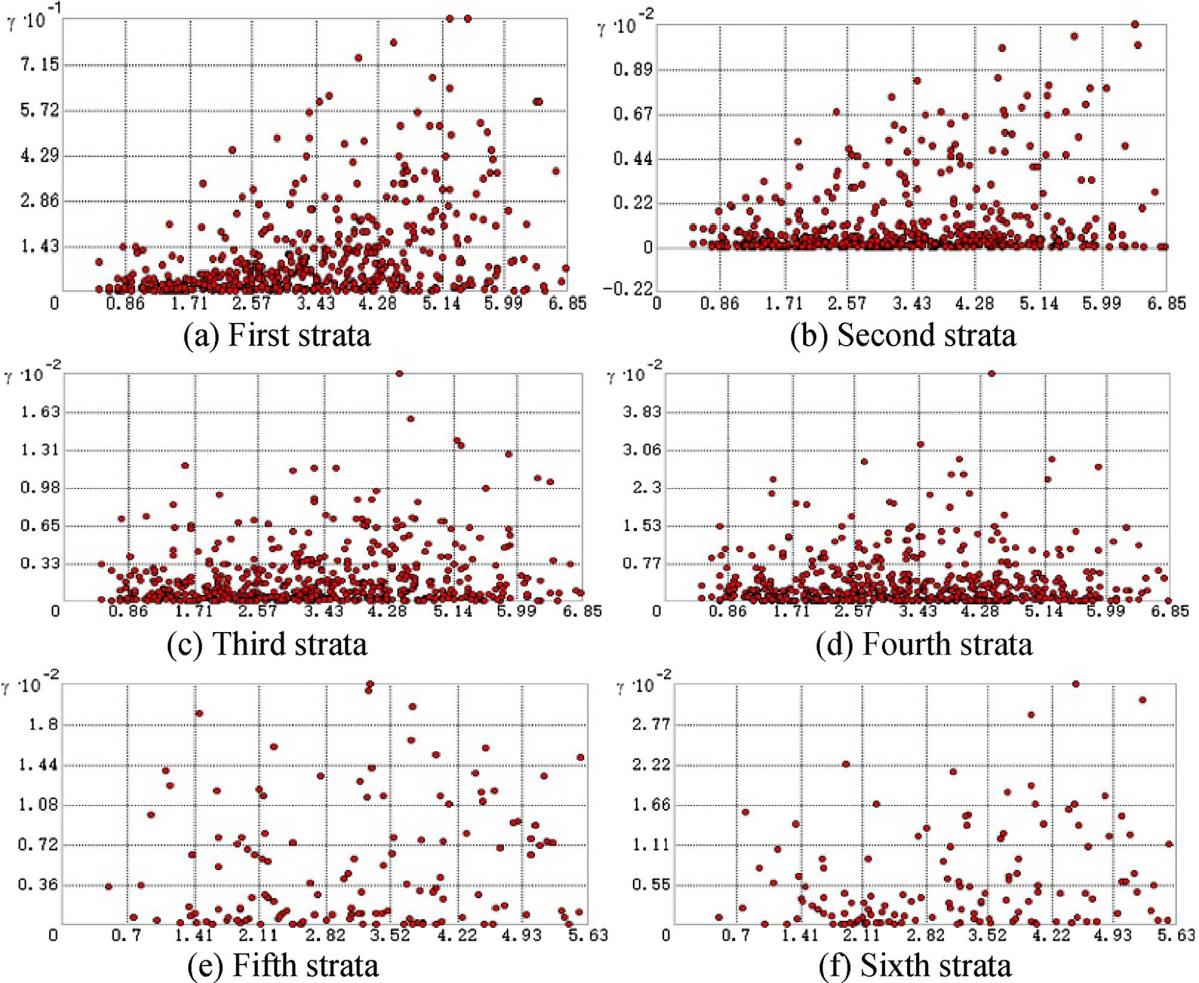
Variation function cloud map of each stratum in which each point represents a data pair, the abscissa represents the distance between the two points, and the ordinate represents the variation function value. The first layer and the second layer show that as the distance of the sample points increases, the value of the variation function increases gradually, and the point cloud increases gradually, which accords with the characteristics of spatial autocorrelation. The cloud images of the other four strata are scattered and have no obvious trend, indicating that they do not have the property of spatial autocorrelation.

After data fusion, the variation function cloud map was used to analyze the spatial autocorrelation of each stratum demarcation point. Figure 13 shows the variation function cloud map of each stratum sampling point.

Because the first stratum and the second stratum were spatially autocorrelated and met the precondition of ordinary Kriging interpolation, four commonly used interpolation algorithms were used to encrypt these strata. The third to the sixth strata were not spatially autocorrelated; and only inverse distance weighting, thin plate splines and regular splines were used for interpolation. The specific interpolation results are shown in Table 1.

**Table 1:** Interpolation results of each stratum.

Stratum	Kriging	IDW	Thin plate spline	Regular spline function
Z0				
Z1				
Z2				
Z3				
Z4				
Z5				

From the interpolation results, it can be seen that the six strata are generally high on the left (west), and low on the right (east). The fluctuations of Z0 and Z1 in the region are slow while the changes of Z2-Z5 are violent. Among the methods, the surface obtained by Kriging interpolation can make a smooth transition between high and low points, and the surface obtained by IDW interpolation has obvious convex hulls and pits near the high and low points. The frequent ups and downs in strata Z2 and Z3 reflect obvious "buphthalmos" phenomena. The interpolation result of the thin plate spline is the smoothest, but the whole interpolation result is high, and there are wrinkles and edge warping in the left corner. The surface of regular spline interpolation is between those of Kriging and inverse distance interpolation. The surface is smooth, but there is still a local "buphthalmos" phenomenon.

In terms of interpolation efficiency, the IDW interpolation only considers the impact of distance changes on the estimation, so the efficiency is the fastest. Kriging interpolation first analyzes the mechanism of the sample data, optimizes the parameters of the variation function, and takes into account the unbiased and optimal conditions; therefore, its calculation is more complex, and the interpolation efficiency is low. As types of functional interpolation, the thin plate spline and regular spline need to solve the undetermined coefficients, and their interpolation efficiency is medium.

Based on the interpolation results of the four algorithms, the leave-one-out method is used to cross-validate the algorithms. Then, the formation degree of fit of interpolation is quantified, and the relative error and root mean square error are selected as the final criteria to measure the accuracy. The specific results are shown in Table 2.

**Table 2:** Cross-validation results.

Stratum	Cross validation	Kriging	IDW	Thin plate spline	Regular spline function
Z0	Average error	0.845	1.177	0.979	0.951
	Root mean square	1.144	1.600	1.251	1.286
Z1	Average error	1.591	2.203	2.584	2.225
	Root mean square	2.132	2.852	3.124	2.809
Z2	Average error	–	3.029	3.136	2.859
	Root mean square	–	3.805	4.110	3.711
Z3	Average error	–	5.442	5.943	5.646
	Root mean square	–	6.523	7.131	6.744
Z4	Average error	–	5.714	9.198	6.419
	Root mean square	–	6.783	10.422	6.657
Z5	Average error	–	5.715	6.328	4.585
	Root mean square	–	5.581	7.503	5.766

As shown in Table 2, the best interpolation results are obtained by selecting Kriging interpolation for strata Z0 and Z1, the inverse distance weighting method for strata Z3 and Z5, and the selection rule spline function for formation Z2. The interpolation result of formation Z4 is unique. The average error of IDW interpolation is the smallest, and the mean square error of regular spline function is the smallest. However, the thin plate spline makes the surface deviate from the actual stratum because of overfitting, and the accuracy is low.

**Figure 14:**
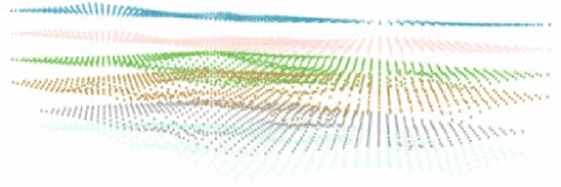
Stratum point model.

**Figure 15:**
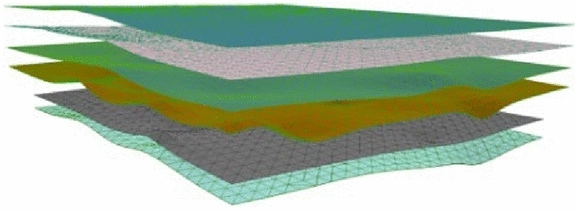
Stratigraphic interface model.

The best interpolation method obtained by analysis and comparison was used to interpolate each stratum at an equal distance, and the layer point model was constructed as shown in Fig. 14. Based on the model, a triangulation was generated to realize the construction of the stratigraphic interface model, and the final result is shown in Fig. 15.

**Figure 16:**
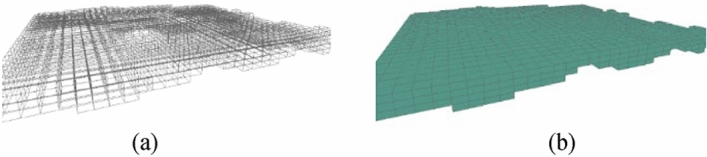
Rendering regular voxels.

The specific graphical steps for constructing the geological body model are as follows:

Step 1: As shown in Fig. 16, we first calculate the position of the regular voxels and their rendered forms.

**Figure 17:**
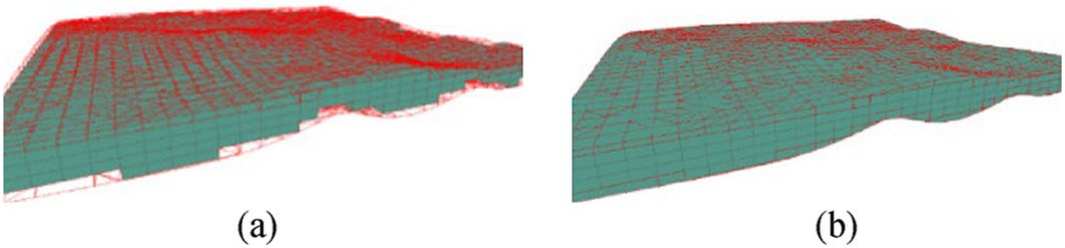
Render irregular voxels.

Step 2: Next, we calculate the shape and position of the irregular voxels and their rendered forms, as shown in Fig. 17.

**Figure 18:**
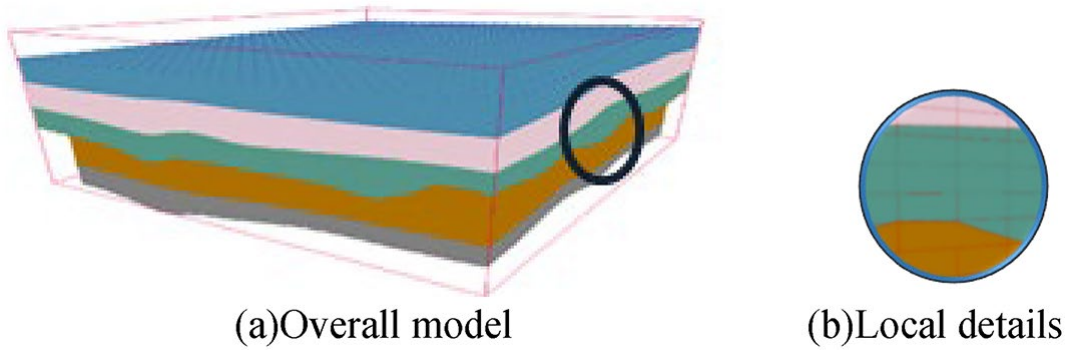
Overall model and local details.

Step 3: Step one and step two were repeated in each stratum to complete the rendering of the whole model. For example, when the regular mesh resolution was set to 200 m × 200 m and the voxel height was set to 4 m, a total of 20,193 voxel metadata points and 96,855 vertex data points were generated. As seen from Fig. 18, the split model can better support the smooth expression of the stratigraphic interface. The total time required for all voxels to complete rendering and loading is approximately 21.98 s. The FPS is maintained at around 60, which maintains good stability while rendering.

**Figure 19:**
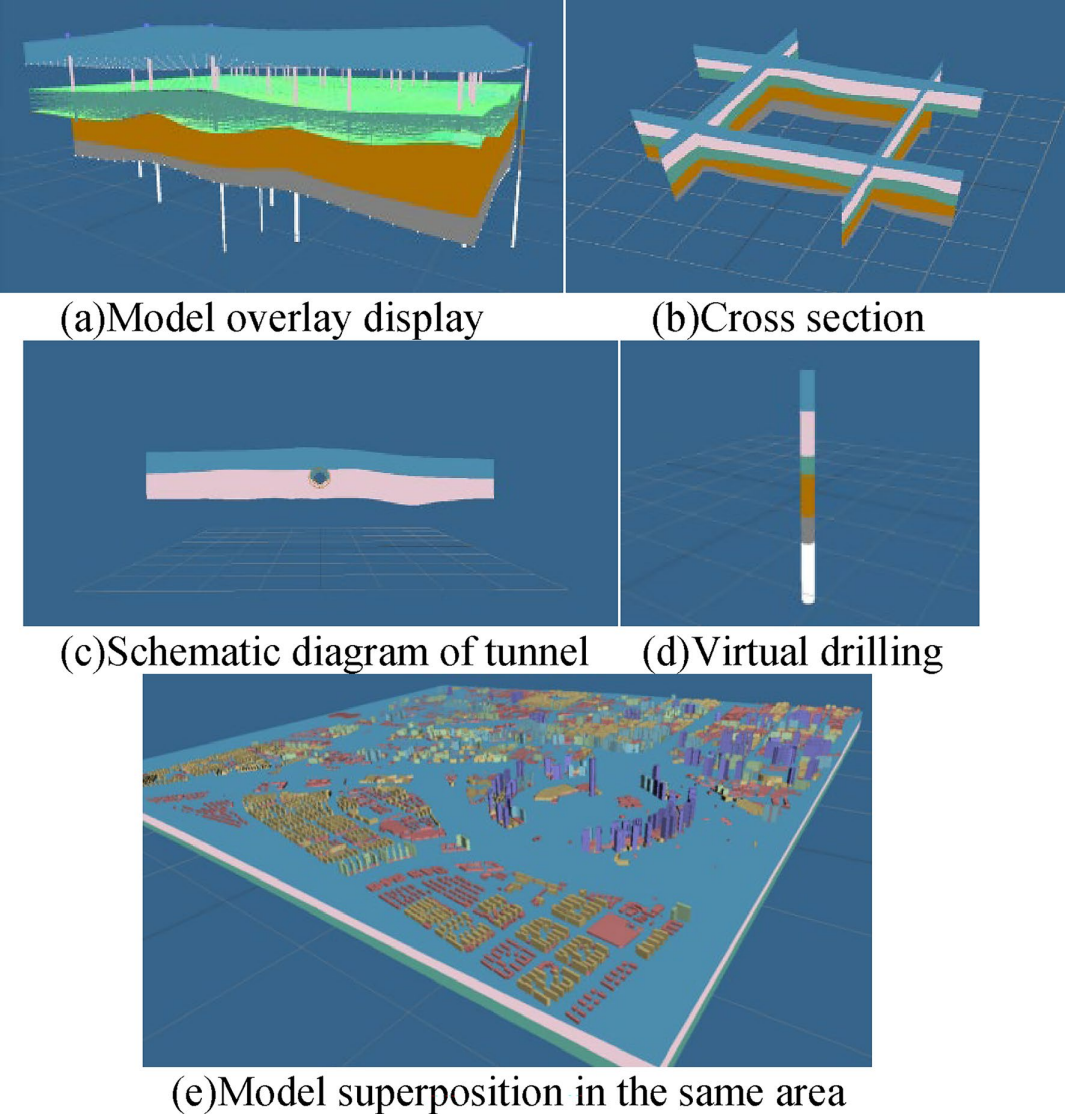
Prototype system model display.

In the prototype system, the practicability of the model was also fully verified. Figure 19a shows the overlay display of all the models; Fig. 19b shows the cross section of the model; Fig. 19c shows the simulated tunnel excavation; Fig. 19d shows the generation of virtual boreholes; and Fig. 19e shows the superposition of the geological model and the building model in the same area, which visually shows the geological structure where the building is located.

## Conclusion and perspectives

In this paper, a regular voxel splitting model based on hierarchical interpolation data was creatively proposed. The foundation of the model is the fusion of borehole and seismic section data and the determination of the best interpolation algorithm. Compared with the previous data fusion methods, the whole process deeply analyzes the original image data and borehole data and unifies the two formats. Then, the algorithm with the best fitting effect was adopted to interpolate and encrypt the fused data, which provided precise data support for subsequent modeling to the maximum extent. On the basis of the regular block, according to the different positional relationships between the voxel and the formation interface, the proposed splitting model solves the defect that the traditional regular voxel cannot describe the boundary smoothly and retains the advantage of attribute expression. In addition, the designed data structure can achieve efficient data access and parsing in the visualization stage of the model. Furthermore, the prototype system based on Three.js eliminates the constraints of desktop applications and web plug-ins on the web side and realizes model construction, 3D display and geological analysis on the browser side without plug-in support. In the later application of urban underground 3D modeling, the model can provide sufficient support for the study of internal information such as groundwater simulation, soil pollution, temperature, pressure, etc. Moreover, we can combine numerical simulation, geotechnical mechanics and other related topics to simulate the impacts of possible urban geological disasters on the surface and other areas.

At present, the voxel split modeling algorithm and the whole workflow proposed in this paper can realize the rapid underground 3D modeling in plain urban areas, but they cannot be well applied to the underground conditions with complex geological structures and violent topographies. In future work, we need to further study the regional implementation of the voxel splitting model so that it can adapt to complex geological conditions such as folds and faults to expand the application scope of the model.
